# Biogenic nanosilver synthesized in *Metarhizium robertsii* waste mycelium extract – As a modulator of *Candida albicans* morphogenesis, membrane lipidome and biofilm

**DOI:** 10.1371/journal.pone.0194254

**Published:** 2018-03-19

**Authors:** Barbara Różalska, Beata Sadowska, Aleksandra Budzyńska, Przemysław Bernat, Sylwia Różalska

**Affiliations:** 1 Department of Immunology and Infectious Biology, Institute of Microbiology, Biotechnology and Immunology, Faculty of Biology and Environmental Protection, University of Lodz, Lodz, Poland; 2 Laboratory of Microbiological and Technical Services, Institute of Microbiology, Biotechnology and Immunology, Faculty of Biology and Environmental Protection, University of Lodz, Lodz, Poland; 3 Department of Industrial Microbiology and Biotechnology, Institute of Microbiology, Biotechnology and Immunology, Faculty of Biology and Environmental Protection, University of Lodz, Lodz, Poland; VIT University, INDIA

## Abstract

Due to low efficacy of classic antimicrobial drugs, finding new active preparations attracts much attention. In this study an innovative, cost-effective and environmentally friendly method was applied to produce silver nanoparticles (AgNPs) using filamentous fungi *Metarhizium robertsii* biomass waste. It was shown that these NPs possess prominent antifungal effects against *C*. *albicans*, *C*. *glabrata* and *C*. *parapsilosis* reference strains. Further detailed studies were performed on *C*. *albicans* ATCC 90028. AgNPs kill curve (CFU method and esterase-mediated reduction of fluorescein diacetate); fractionally inhibitory concentration index (FICI) with fluconazole (FLC); effect on fungal cell membrane permeability (propidium iodide (PI) staining), membrane lipids profile (HPLC-MS), yeast morphotypes and intracellular reactive oxygen species level (H_2_DCFDA probe) were investigated. Anti-adhesive and anti-biofilm properties of AgNPs (alone and in combination with FLC) were also tested. Biosafety of AgNPs use was assessed *in vitro* in cytotoxicity tests against L929 fibroblasts, pulmonary epithelial A549 cell line, and red blood cells. Significant reduction in the viability of yeast cells treated with AgNPs was shown within 6 h. The proportion of *C*. *albicans* PI-positive cells increased in a dose and time-dependent manner. Changes in the qualitative and quantitative profile of cell membrane lipids, including significant decline in the quantity of most phospholipid species containing C18:2 and an increase in the amount of phospholipids containing C18:1 acyl species were observed after yeast exposure to AgNPs. CLSM images showed an enhancement in ROS intracellular accumulation in *C*. *albicans* treated with biogenic nanosilver. *C*. *albicans* transformation from yeast to hyphal forms was also reduced. AgNPs decreased adhesion of yeast to abiotic surfaces, as well as acted synergistically with FLC against sessile population. At fungicidal and fungistatic concentrations, they were non-toxic to mammalian cells. Obtained results confirm suitability of our “green synthesis” method to produce AgNPs with therapeutic potential against fungal infections.

## Introduction

The treatment of infectious diseases remains an important and challenging medical problem due to the combination of factors, such as new infections emerging, an increasing population at risk, and a growing number of multi-drug resistant pathogens. Thus there is an urgent need to explore therapeutic options using new antimicrobial agents, possibly those acting by mechanism distinct from that of current therapeutics. Investigations involve mainly searching for substances possessing direct antimicrobial activity and/or can synergise with classic pharmacological agents. Recent directions, however, have also looked for products that can restrict the expression of attributes in microbial virulence or activate host immune defence mechanisms [1–5]. In some cases, newly discovered products have shown several such properties.

Metal ions, their nanoparticles and complexes with various ligands, promise a new therapeutic perspective discussed above, and are now attracting considerable attention. Numerous metals have been used since ancient times as antimicrobial agents. In history there are many applications of copper (Cu), silver (Ag), tellurium (Te), magnesium (Mg) and arsenic (As) oxides, as well as Cu and mercury (Hg) salts, to treat severe diseases of wide epidemiological and social importance. Although the recent focus is on metal nanoparticles (NPs), intensive study is being conducted all over the world and the *in vitro* findings are already extensive, nevertheless the further search for the most effective agents in the fight against pathogens is needed, as well as a new perspective [6–9].

Metal nanoparticles for the abovementioned purposes are usually produced mostly by chemical or physical methods that help to make particles with the required properties, but unfortunately they are associated with the use of hazardous chemicals. Thus, it is desirable to develop alternative non-toxic, environmentally harmless and cheap procedures in metal NPs production. Among several approaches, biogenic synthesis is attracting more attention as a clean, efficient and environmentally safe method. For this type of synthesis, culture filtrates, bio-extracts from bacteria, plants or fungi can act as reducing and capping agents [10–14]. It is known that such parameters as nanoparticles size, shape, crystalline structure and many others dependent on the production method, impact on their antimicrobial effectiveness. Thus, it was necessary not only develop an innovative, cost-effectively and eco-friendly method to produce nanosilver but also to assess antimicrobial activity and biosafety of the final product to prove the hypothesis of their therapeutic and application potential. We have already reported that the extract of waste biomass obtained from cultures of the filamentous fungus, *Metarhizium robertsii*, following nonylphenol biodegradation can generate small, homogenous in size and predominantly spherical silver nanoparticles (AgNPs) [15]. This product possesses significant antimicrobial activity against Gram-positive and Gram-negative bacteria; nevertheless, its ability to interfere with fungal pathogens has yet to be thoroughly investigated. Therefore we used *Candida albicans* yeast as the final target microorganism of biogenic nanoscale silver. Epidemiological data shows that it is the fungal species most frequently isolated from clinical samples, justifying its selection for further study [16].

Changes in the expression of many *C*. *albicans* genes in different types of infections in a specific host niches and different stages of disease can be detected. However, the activity of numerous adhesins, of tissue-damaging hydrolytic enzymes, the ability to transform morphologically between yeast and hyphal forms, and efficient evasion of host immune cells, are its most important virulence factors. A widely accepted view supported by clinical observation is that, like bacteria, the majority of fungi (including *Candida* species) can form free-floating aggregates and/or biofilms attached to abiotic or living surfaces. Clusters of fungal cells become embedded in a self-produced extracellular polymeric substance (EPS) that forms a barrier against antimicrobial agents. A number of other complementary factors contribute to biofilm drug resistance, such as diverse metabolic activity of the cells located at different layers of the structure (the gradients of oxygen and nutrients force the stratification of microbes in a biofilm), increased cell-associated efflux pump activity, horizontal transfer of resistance genes, among others [16–18]. Since biofilm resistance to classic therapeutics is high, several approaches to overcome this obstacle have been proposed. We present data herein on antifungal/anti-biofilm activity of AgNPs produced using an innovative, cost-effectively and environmentally friendly method in waste culture extracts of *M*. *robertsii*, by showing their influence on the pathogenic potential of *Candida* cells.

## Materials and methods

### Characteristics of biogenic nanoscale silver

During the preliminary experiments different biomass extract contents (25, 50, 75 and 100%) were tested. The highest reduction rates were noted for diluted samples (25%). Another parameter—photoinduction time, was experimentally determined at 3 h as it was described by Różalska et al. [15]. Obtained AgNPs were small, homogenous in size and predominantly spherical under such conditions of synthesis. Other experimental conditions e.g. higher temperature (25, 28, 30°C) did not have positive influence on synthesized AgNPs. The colloids were stable in PBS buffer and RPMI-1640 medium for four weeks, while in water—longer than two months. Finally, the AgNPs synthesis procedure was set as follows: 25% biomass extract (v/v in deionized water) free from mycelium was filter sterilized and a freshly prepared aqueous solution of silver nitrate was added to a final concentration of 1 mM. The samples were incubated under bright conditions with radiation (1800 μmol/m^2^/s) for 3 h [15].

### Target microorganisms, growth media and culture conditions

The reference strains—*C*. *albicans* ATCC 10231, *C*. *albicans* ATCC 90028, *C*. *glabrata* ATCC 90030 and *C*. *parapsilosis* ATCC 22019, grown for 48 h at 35°C on Sabouraud Dextrose Agar (SDA)—were used to screen the anti-fungal activity of AgNPs. Dependent on the test, yeast suspensions at 5×10^5^ to 10^6^ cells/mL were prepared in the RPMI-1640 medium.

### AgNPs Minimal Inhibitory (MIC) / Fungicidal Concentration (MFC)

The MIC and MFC of AgNPs (0.39–50 mg/mL) filtered through membrane filter with pore size 0.45 μm (Minisart, Sartorius) were determined using the microdilution broth assay recommended by EUCAST [19]. Briefly, MIC measured spectrophotometrically (Victor2 Wallac, Finland) was defined as the lowest concentration of AgNPs inhibiting fungal growth after 24 h co-incubation at 37°C compared to the appropriate positive controls. MFC means the lowest concentration of the NPs that prevent the growth of the yeast after subculturing 10 μL from the wells marked as MIC, 2× MIC and 4× MIC, on SDA medium free of NPs and further incubation for 24 h at 37°C. MIC of the reference drug—fluconazole (FLC, at 0.25–64 μg/mL) was determined by the same protocol. Experiments were carried out in triplicate on two separate occasions.

### Fractionally inhibitory concentration index (FICI) of AgNPs combined with fluconazole

The antifungal combination against planktonic *C*. *albicans* ATCC 90028 (at 5×10^5^ cells/mL) was assessed by mixing serial dilutions of AgNPs (0.39–6.25 μg/mL; MIC = 6.25 μg/mL) and FLC (0.06–1.0 μg/mL; MIC = 1.0 μg/mL). The MICs of the combination of FLC + AgNPs were determined spectrophotometrically after 24 h incubation at 37°C (Victor2 Wallac, Finland). The FICI was calculated in this checkerboard dilution assay by the formula:

$$FICI=FIC\;A+FIC\;B=\frac{\left[A\right]}{MIC\;A}+\frac{\left[B\right]}{MIC\;B}$$ (1)

where [A] is the concentration of drug A, MIC A is its MIC, and FIC A is the FIC of drug A for the organism, whereas [B], MIC B, and FIC B are defined in the same fashion for drug B. The FICI data was interpreted as follows: <1 means synergy; >1 to 4 means indifference, and >4 means antagonism [20]. The test was run twice in triplicate.

### *C*. *albicans* time-kill and cell membrane permeability

A suspension of *C*. *albicans* ATCC 90028 at 5 × 10^5^ cells/mL in RPMI-1640 medium was cultured in the presence or absence of AgNPs at 0.5× MIC or MIC for 24 h at 37°C. At the chosen time-points (1, 2, 4, 6, 8, 24 h) 100 μL of the samples were serially diluted in 0.85% NaCl, spread on SDA plates and cultured (48 h at 37°C) to count the colony forming units (CFU), the results being expressed as a mean number of CFU ± S.D. calculated from 4 replicates of two independent experiments.

In parallel, the metabolic activity of viable cells was measured using a modified "FDA" (fluorescein diacetate) method [21, 22]. FDA is a cell-permeant esterase substrate that serves as a viability probe, measuring both enzymatic activity (required to activate FDA fluorescence) and cell-membrane integrity, necessary for intracellular retention of its fluorescent product. Briefly, 200 μL *C*. *albicans* culture samples at 2, 4, 6 h of co-incubation with AgNPs were put into 96-well plates (Nunc, USA) and 100 μl FDA solution (0.2 mg/mL in 0.1 M phosphate buffer, pH = 6.4) was added (per well) for a 1 h incubation at 37°C in the dark. The fluorescence was read at an excitation of 485 nm and emission of 520 nm (SpectraMax i3, Molec. Devices). The results were expressed as a percentage of enzyme (esterase) activity calculated compared to the RFU (relative fluorescence units) between tested and control yeast cells.

The action of AgNPs on the yeast membrane permeability was measured by propidium iodide exclusion (PI, Sigma, USA). Aliquots of the samples removed after 2, 6, 24 h AgNPs treatment were centrifuged, washed with PBS and suspended in 100 μL PI solution (60 μM in H_2_O) for 20 min in the dark at room temperature. "Stained" samples washed with PBS were analyzed microscopically (Confocal Laser Scanning Microscope, LSM 510 Meta, Zeiss, Germany) with a He–Ne laser (543 nm) and a LP filter set (560–615 nm), and Nomarski DIC set at an excitation of 543 nm. The total area of yeast cells and areas emitting red fluorescence were assessed. Based on the data, the percentage of cells that fluoresced (were damaged or dead) was calculated. Representative images of control and tested samples are shown, and image analyses of the data described in the text.

### *C*. *albicans* cell membrane lipid profile

The following reagents were used: 1,2-dimyristoyl-sn-glycero-3-phospho-rac-(1-glycerol) (sodium salt) (14:0/14:0 PG); 1,2-dilauroyl-sn-glycero-3-phosphoethanolamine (12:0/12:0 PE); 1,2-dimyristoyl-sn-glycero-3-phosphocholine (14:0/14:0 PC); 1,2-dipalmitoyl-sn-glycero-3-phospho-(1′-myo-inositol) ammonium salt (16:0 PI) sodium salt from bovine liver; 1,2-dimyristoyl-sn-glycero-3-phospho-L-serine sodium salt (14:0/14:0 PS); and 1,2-dimyristoyl-sn-glycero-3-phosphate (sodium salt) (14:0/14:0 PA) purchased from Avanti Polar Lipids (Alabaster, AL, USA). Each of the compounds was added to a solution of mixed internal standards at 0.1 μg/mL (IS solution). The other chemicals were purchased from JT Baker (St. Louis, MO, USA), Fluka (Poznan, Poland), and POCh (Gliwice, Poland). All chemicals were of high purity grade.

Phospholipids (PLs) from cultures of *C*. *albicans* were extracted as per Folch et al. [23], with some modifications. The yeast biomass was transferred into Eppendorf tubes containing glass beads, 0.66 mL chloroform and 0.33 mL methanol, and then yeast cells were disrupted by vigorous shaking in a Fastprep-24 homogenizer for 1 min. The mixture was extracted by vortexing for 2 min. Separation of 2 layers was facilitated by adding 0.2 mL 0.9% saline. The lower layer was collected and evaporated. Polar lipids were measured using an Agilent 1200 HPLC system (Santa Clara, CA, USA) and a 4500 Q-TRAP mass spectrometer (Sciex, Framingham, MA, USA) with an ESI source. For the reversed-phase chromatographic analysis, 10 μL lipid extract was injected onto a Kinetex C18 column (50 mm × 2.1 mm, particle size: 5 μm; Phenomenex, Torrance, CA, USA). The mobile phase consisted of 5 mM ammonium formate in water (A) and 5 mM ammonium formate in methanol (B). The solvent gradient started at 70% B, increased to 95% B over 1.25 min, and was maintained at 95% B for 6 min before returning to the initial solvent over 3 min. The column temperature was maintained at 40°C with a flow-rate of 500 μl/min. Prior to the next injection, a blank sample of methanol was run. The instrument settings were as follows: spray voltage −4.500 V, curtain gas (CUR) 25, nebulizer gas (GS1) 50, turbo gas (GS2) 60, and the ion source temperature 600°C. Data analysis used Analyst™ v1.6.2 software (Sciex, Framingham, MA, USA). Two approaches were applied to identify PLs: targeted and untargeted. The untargeted approach was performed with the precursor ion scanning (precursor for *m/z* 153) survey scan, triggering the EPI experiments. On the basis of the untargeted analysis, a comprehensive list of the multiple reaction monitoring (MRM) transitions was generated (parent fatty acyl fragment) for the following PL classes: PA, PC, PE, PS, Cl and PI.

### *C*. *albicans* micromorphology

To follow the kinetics of morphogenesis, *C*. *albicans* ATCC 90028 suspension at initial density of 8 × 10^6^ cells/mL was incubated (for 1–3 h at 37°C) in RPMI-1640 medium containing 10% (v/v) of fetal calf serum (FBS, Cytogen, Poland) without (control) or with the addition (1:1) AgNPs at 0.5× MIC or MIC. The fraction of germ tube form (GTF) positive cells against budding blastospores and blastospores with unchanged morphology was assessed by light microscopy (Nikon Eclipse E200, 400× magnification). The criteria for cell morphotype evaluation were as follows: germination means that the cell had a germ tube at least twice the cell diameter; and budding means that the cell had a bud at least one half the diameter of the mother cell. The results were expressed as the proportion (± S.D.) of each morphotype, calculated from 500 cells, after AgNPs treatment compared to control *C*. *albicans*. After prolonged incubation time (an additional 24 h), hyphal morphology was assessed microscopically in each culture.

Detailed cell/hypha morphology was visualized by staining with ready-to-use Calcofluor White solution (Sigma, USA). Briefly, 10 μL samples (test, control) taken at selected time- points were mixed with dye (1:1) on a slide. Samples were assessed microscopically (fluorescence microscope, Zeiss, AXIO Scope A1, magnification 400×) and representative images taken.

### Intracellular reactive oxygen species (ROS) levels in *C*. *albicans*

ROS levels in *C*. *albicans* cells were measured after 2, 6, 24 h incubation of yeasts at 1 × 10^6^ cells/mL with AgNPs at 0.5× MIC or MIC, using 2',7'-dichlorodihydrofluorescein diacetate (H_2_DCFDA, Sigma-Aldrich, Germany). Briefly, the control and test samples were centrifuged, washed with PBS and suspended in 200 μL H_2_DCFDA working solution, which had been empirically determined as 40 mM H_2_DCFDA in DMSO, 20× diluted in PBS, for a 15 min incubation in the dark at room temperature. The cell-permeant H_2_DCFDA detects ROS in the cells by cleavage of the acetate groups by intracellular esterase and oxidation non-fluorescent; H_2_DCFDA is converted to highly fluorescent 2',7'-dichlorofluorescein. After washing with PBS, the samples were analyzed by Confocal Laser Scanning Microscope (LSM 510 Meta, Zeiss, Germany). Representative images of fields in control and tested samples were taken. The total area of fungal cells and areas emitting green fluorescence were measured, from which the percentage of cells producing ROS was calculated.

### *C*. *albicans* adhesion, biofilm formation, and its eradication under the influence of AgNPs

*C*. *albicans* ATCC 90028 suspension at 1 × 10^6^ cells/mL in RPMI-1640 medium supplemented with 0.25% glucose were seeded (100 μL) into the wells of 96-well polystyrene microtiter plates (Nunc, Denmark). AgNPs at 0.5× MIC (100 μL) were added for 2 h (to measure adhesion) and 24 h (to measure biofilm formation) at 37°C in static conditions. After incubation for different times, non-adherent cells were removed by washing and the metabolic activity of the sessile population measured using the modified FDA method described above. Briefly, 100 μL FDA solution (0.2 mg/mL in phosphate buffer at pH 6.8) was added for 1 h incubation at 37°C in the dark and the fluorescence emitted read at 485/520 nm in the SpectraMax i3. The results are presented as the percentage of adherent cells or total biofilm mass metabolic activity, calculated from the values of FDA reduction (RFU, relative fluorescence units) ± S.D. of the test wells compared to the controls taken as 100%.

To determine the MBEC_50_ of the AgNPs—the concentration that "inhibits" 50% of biofilm biomass—FDA and the Alamar Blue reduction assays were used. NPs were added to a 24 h biofilm at concentrations ranging from MIC to 16× MIC (6.25–100 μg/mL). Both methods were used to measure intracellular metabolic activity, but at different subcellular levels. The same protocol of biofilm staining with FDA was used as described above, whereas the Alamar Blue reduction test was done as indicated by the manufacturer (Invitrogen, UK). MBEC_50_ of the reference drug, fluconazole, used at a concentration range from MIC to 256× MIC (1.0–256 μg/mL), was determined using the same protocol. The results were estimated as the percentage of total biofilm mass metabolic activity calculated from the values of FDA/Alamar Blue reduction ± S.D. of the control (taken as 100%). The experiments were repeated twice in triplicate.

### Anti-biofilm action of AgNPs when combined with fluconazole

Fluconazole was used on 24 h- old *C*. *albicans* biofilms alone or together with AgNPs, both agents at different combinations of concentrations pre-determined for planktonic or sessile population, which were as follows: the first set contained MIC_FLC_ + MIC_AgNPs_; + 2× MIC_AgNPs_; + 4× MIC_AgNPs_; and the second set contained MBEC_50 FLC_ + MIC_AgNPs_; + 2× MIC_AgNPs_; + 4× MIC _AgNPs_. After the incubation for a further 24 h at 37°C, "biofilm” was stained by the same protocol as described above. Briefly, the minimal biofilm eradication concentration of the drug combination, which decreased metabolic activity of the biomass by >50% (>MBEC_50_), was measured using the Alamar Blue reduction assay as recommended by the manufacturer. The experiments were repeated twice in triplicate.

### Cytotoxic activity of AgNPs against fibroblasts and epithelial cells

L929 fibroblasts (ATCC, CCL 1) and A549 lung epithelial cell line (ATCC, CCL 185) were grown in RPMI-1640 medium with L-glutamine and sodium bicarbonate (Sigma, USA) supplemented with 10% (v/v) heat-inactivated FBS (Cytogen, Poland) and 1% (v/v) penicillin/streptomycin (Sigma, USA), at 3°C in a humidified atmosphere of air with 5% CO_2_ for 3 days. Confluent monolayers of both cell types were detached by scrapping, suspension of 1 × 10^5^ cells/mL prepared and 100 μL (per well) seeded into 96-well tissue culture plates (Nunc, Denmark) for 24 h incubation at 37°C. The culture medium was replaced with 200 μL medium containing the AgNPs at 0.19–25 μg/mL for 2 and 24 h. Appropriate positive and negative controls were set up. The cytotoxicity of the AgNPs was measured by MTT reduction assay, as before [24]. Briefly, the microplates were centrifuged (1400 rpm, 10 min) and washed with PBS supplemented with Ca^2+^/Mg^2+^. The supernatants were removed and 100 μL fresh culture medium and 50 μL MTT in PBS (1.5 mg/mL) were added to the wells, which was followed by 2 h incubation in the dark. The medium with MTT was discarded and 75 μL 20% (w/v) SDS (sodium dodecyl sulfate; Sigma) in a mixture of dimethylformamide with water (1:1 ratio) was added for 18 h incubation at room temperature in the dark. Absorbance of the samples was read at 550 nm using a microplate reader (Victor2; Wallac, Finland). The results for the test samples and the controls were used to calculate the percentage of cell viability and the IC_50_ (concentration giving 50% loss of viability).

### Hemolytic activity of AgNPs against human red blood cells

Pooled human blood sample (sodium citrate diluted) was purchased from Regional Blood Center in Lodz, Poland. Hemolysis was assayed using the standard protocol. Briefly, blood was centrifuged (2400 rpm, 5 min, +4μC), washed with PBS and the red cells (hRBC) pellet was suspended in PBS to adjust it to 4% (v/v). An AgNPs dilution series in PBS (the final concentration 0.19–25 μg/mL), 100 μL PBS as a blank, and 100 μL of Triton-X-100 (Sigma-Aldrich, USA) as positive control, were added to 100 μL hRBC suspension in U-bottomed microplates. Following incubation for 2 and 24 h at 37°C, the mixture was centrifuged (1200 rpm, 10 min) and 100 μL supernatant transferred to each well of new 96-well plates. Absorbance (OD) at 490 nm was determined in the microplate reader. The percentage of hemolysis was calculated by the equation:

$$\%\;hemolysis\;(H)=\left[\frac{H\;sample-H\;blank\;control}{H\;positive\;control-H\;blank\;control}\right]\;x\;100\%$$ (2)

### Statistic evaluation

Most of the results are given as the mean values with standard deviation (S.D.). When applicable, statistical differences between treated and control groups were assessed by the Mann-Whitney *U* test using STATISTICA 12.0 (Stat Soft Inc., USA) software. *P*≤ 0.05 was taken as statistically significant.

## Results and discussion

We show strong antifungal activity of nanosilver particles obtained from AgNO_3_ by an eco-friendly bottom-up technique using an extract from waste biomass of entomopathogenic fungi *Metarhizium robertsii* culture, as described by Różalska et al. [15]. Basic parameters of these nanoparticles were previously done by UV-VIS spectroscopy and DLS measurements. The used colloid surface plasmon resonance band maximum was detected at 465 nm and the polydispersity index (PDI) value was 0.262. TEM analysis showed that AgNPs were mostly spherical in shape of size from 15 to 25 nm. AgNPs were tested for antibacterial properties against *Staphylococcus aureus* and *Escherichia coli* (MIC values were 30 and 15 μg/mL, respectively) [15]. Propidium iodide staining and confocal laser scanning microscopy (CLSM) analysis showed red-stained bacterial cells, indicating that cell membrane damage has occurred [15]. However, antibacterial activity of nanosilver is in fact mediated by multifaceted mechanisms, probably including cell wall synthesis, membrane transport, electron transport in respiratory chain, protein function, as well as DNA transcription and translation, but is not yet fully explained. Franci et al. [14] have summarized the results of research on alternative ways to fight infections of different etiology using silver nanoparticles. Emmanuel et al. [13] reported similar antibacterial potential of AgNPs produced in "green synthesis", as had Różalska et al. [15].

*M*. *robertsii* in our hands as an AgNPs producer is a fungi with multifunctional life styles. It is a saprobiont or colonizer of plant or insect hosts, and is an endophyte known as the ingredient in numerous FDA-approved crop protection formulations [25]. *Metarhizium* spp. produces secondary metabolites that are toxic to the insects and microbes [26]. The results of our study, however, show that *M*. *robertsii* culture is also a source of the metabolites that can participate in extracellular AgNPs synthesis, and that they have a significantly stronger antifungal effect than previously described antibacterial action [15]. The novelty of our study is that AgNPs can be obtained using an eco-friendly method that is also cost-effective. They were synthesized using an extract of fungi waste biomass recovered after degradation of nonylphenol, which has attracted attention due to its bioaccumulation and potential hazardous role as an endocrine disruptor and xenoestrogen [22].

AgNPs produced by *M*. *robertsii* possess prominent antifungal effects against *C*. *albicans*, *C*. *glabrata* and *C*. *parapsilosis* reference strains, with a relatively low MIC range of 1.56–6.25 μg/mL (Table 1).

**Table 1:** Minimal inhibitory concentration (MIC) and minimal fungicidal concentration (MFC) of silver nanoparticles (AgNPs) against *Candida* sp.

*Candida* sp.	AgNPs [μg/mL]	
	MIC	MFC
***C*. *albicans* ATCC 10231**	1.56	1.56
***C*. *albicans* ATCC 90028**	6.25	6.25
***C*. *glabrata* ATCC 90030**	3.12	3.12
***C*. *parapsilosis* ATCC 22019**	6.25	12.50

Note, however, that due to a lack of standardization the antimicrobial activity of nanosilver identified in many other publications is generally inconsistent. The literature data indicate that the MIC values are largely dependent on the method of NPs synthesis, their physico-chemical properties, and the density of the target microbial inoculum. An overview of these reports shows that MICs depend not only on the factors mentioned above, but on the methods used for MIC evaluation. Sets of such methods as agar-disk diffusion, agar well-diffusion, agar dilution or broth macro- or micro-dilution, are characterized by different levels of sensitivity; not all of them are always appropriate for the intended purpose. Similarly, any comparison of the results of different studies is complicated by mentioned differences in the number of cells in the bacterial or fungal target inoculum. Their final density ranges from 10^2^ to 10^5^ cells/mL, as reflected in the activity of the preparations tested [27, 28]. This variation is due to the use of the recommendations of different standardization bodies, such as CLSI or EUCAST, or even the arbitrary nature of the test conditions in any given laboratory. In our opinion, standardized methods established to test antimicrobial activity of these drugs should be used to assess pharmacological potential of the products, which are to replace or support the existing antimicrobial drugs. With this in mind, our findings obtained under restrictive test conditions established by EUCAST [19] indicate that our biogenic silver nanoparticles can have valuable therapeutic potential.

**Fig 1:**
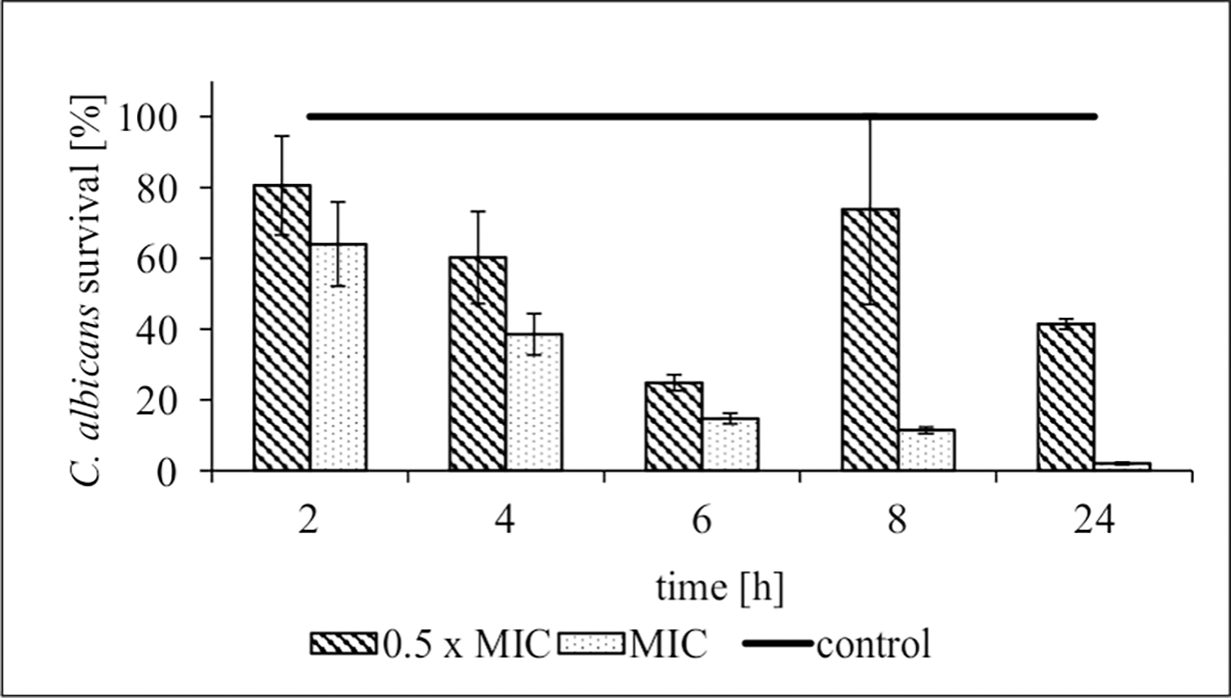
Time and concentration-dependent effect of AgNPs on *C*. *albicans* ATCC 90028 growth. The results are the mean (% of yeast survival ± S.D.) from 2 independent experiments done in quadruplicate as described in Materials and Methods.

Kinetics study showed that after exposure of *C*. *albicans* ATCC 90028 strain (selected for further thorough research) to AgNPs at MIC, the number of viable cells quickly decreased (Fig 1). The number of yeasts after 6 h exposure that could grow on SDA dropped by ~85%. Similar, but a definitely weaker fall in cell viability was also caused by AgNPs at 0.5× MIC. Furthermore, in this case only a transient inhibitory effect of the NPs was recorded over a 2 to 6 h period of co-incubation. But already in the 8th h, cells recovered vitality and their density increased up to 74% of the control level. The number of control cells after 24 h culture reached 884.0 ± 1.0 × 10^5^ CFU (196× increase of the initial inoculum), whereas in culture with AgNPs at MIC or 0.5× MIC, the final cell number was 8.1 ± 2.8 × 10^5^ CFU and 366.0 ± 12.7 × 10^5^ CFU, respectively. In summary, the fungicidal end-point of AgNPs at MIC was reached by 24 h.

**Fig 2:**
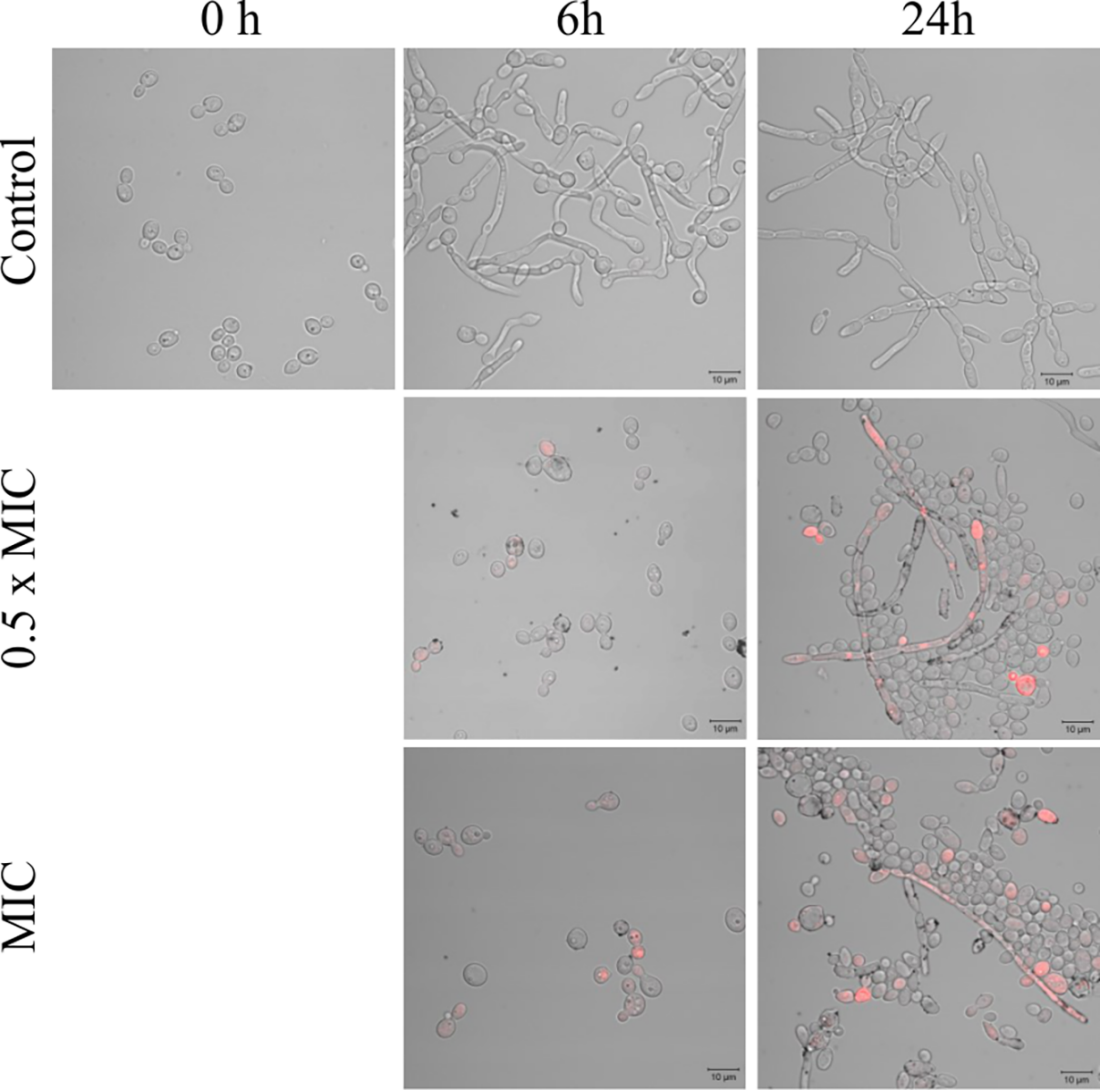
Propidium iodide (PI) staining of *C*. *albicans* ATCC 90028 exposed to AgNPs for 6 and 24 h. Stained samples were analyzed microscopically. Red fluorescence indicates cells with impaired cell membrane permeability. AgNPs attached to the *C*. *albicans* morphotypes are visible as black dots.

The question was asked as to the reason for such unexpected strong effect of sub-MIC temporarily lowering cell ability to grow. This question prompted us to investigate the mechanisms of action of these nanoparticles, such as their effects on membrane permeability, lipid profile and generation of oxidative stress in target cells. Indeed, yeast cells exposed to AgNPs at MIC, and in a lesser extent to 0.5× MIC, suffered significant changes in cell size and granularity. A fraction of the cells stained positively with propidium iodide increased over the control background at 6 h (6 ± 2% and 48 ± 8% for 0.5× MIC and MIC, respectively) and at 24 h of incubation (12 ± 7% and 32 ± 2% for 0.5× MIC and MIC, respectively), which was caused by disturbances in the integrity of the membrane (Fig 2). Similar, very detailed observations have been published by Lara et al. [29].

**Fig 3:**
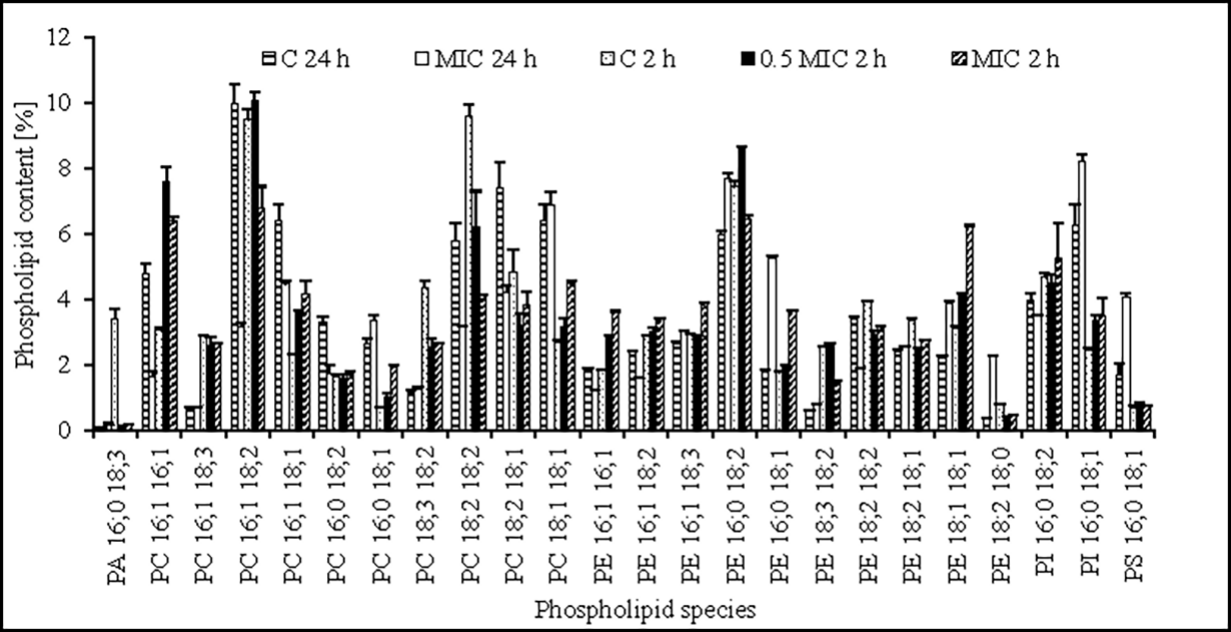
Composition of the main phospholipid species in cell membrane of control *C*. *albicans* ATCC 90028 (C) or treated with AgNP at MIC or 0.5× MIC for 2 and 24 h (measured by HPLC-MS, see Materials and Method). The abundance of lipid species is shown as their percentage in relation to total lipids. PS—phosphatidylserine, PI—phosphatidylinositol, PG—phosphatidylglycerol, PE—phosphatidylethanolamine, PC—phosphatidylcholine, PA—phosphatidic acid.

**Fig 4:**
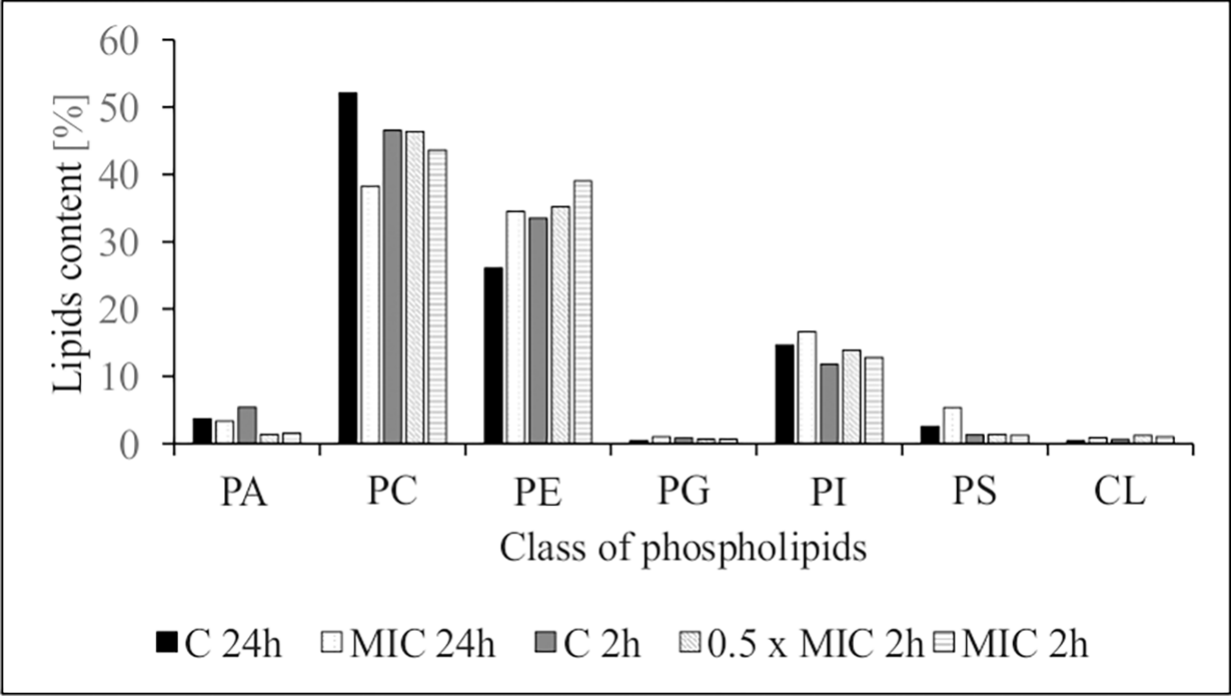
Percentage composition of cell membrane phospholipid classes (Pls) of control *C*. *albicans* ATCC 90028 (C) or cells treated with AgNP at MIC or 0.5× MIC for 2 and 24 h (measured by HPLC-MS). PA—phosphatidic acid, PC—phosphatidylcholine, PE—phosphatidylethanolamine, PG—phosphatidylglycerol, PI—phosphatidylinositol, PS—phosphatidylserine, CL—cardiolipin.

To investigate whether this effect resulted from changes in the profile of lipids in the cell membrane a qualitative and quantitative tests were conducted. Phospholipid (PL) species—the main lipid group constituent of the cell membranes—were identified in *C*. *albicans* ATCC 90028 (Fig 3), and had a profile which was in agreement with that reported elsewhere [30]. Yeast exposed to AgNPs had significantly higher levels of phosphatidylethanolamine (PE) whereas the controls had higher levels of phosphatidylcholine (PC) (Fig 4). Because PE has a strong propensity to form non-bilayer hexagonal phases and PC is a bilayer-stabilizing lipid, the PC/PE ratio is key in both membrane integrity and cell function [31, 32]. Cells exposed to nanoparticles were significantly less in quantity regarding most phospholipid species containing C18:2, and had increase levels of phospholipids containing C18:1 acyl species. Thus, changes in the desaturation rate of fatty acid C18:2Δ^9,12^/C18:1Δ^9^ showed that Δ^12^ desaturation efficiency was inhibited by nanosilver. A specific role of polyunsaturated fatty acids (PUFA) in *C*. *albicans* morphogenesis and membranes fluidity has been reported [33]. Moreover, increased membrane fluidity caused by silver nanoparticles was noted by Park et al. [34] in phospholipid liposomes. Therefore, it cannot be excluded that modifications in fatty acids of *C*. *albicans*, and a decrease in fatty acids unsaturation compensated for the presence of nanoparticles.

**Fig 5:**
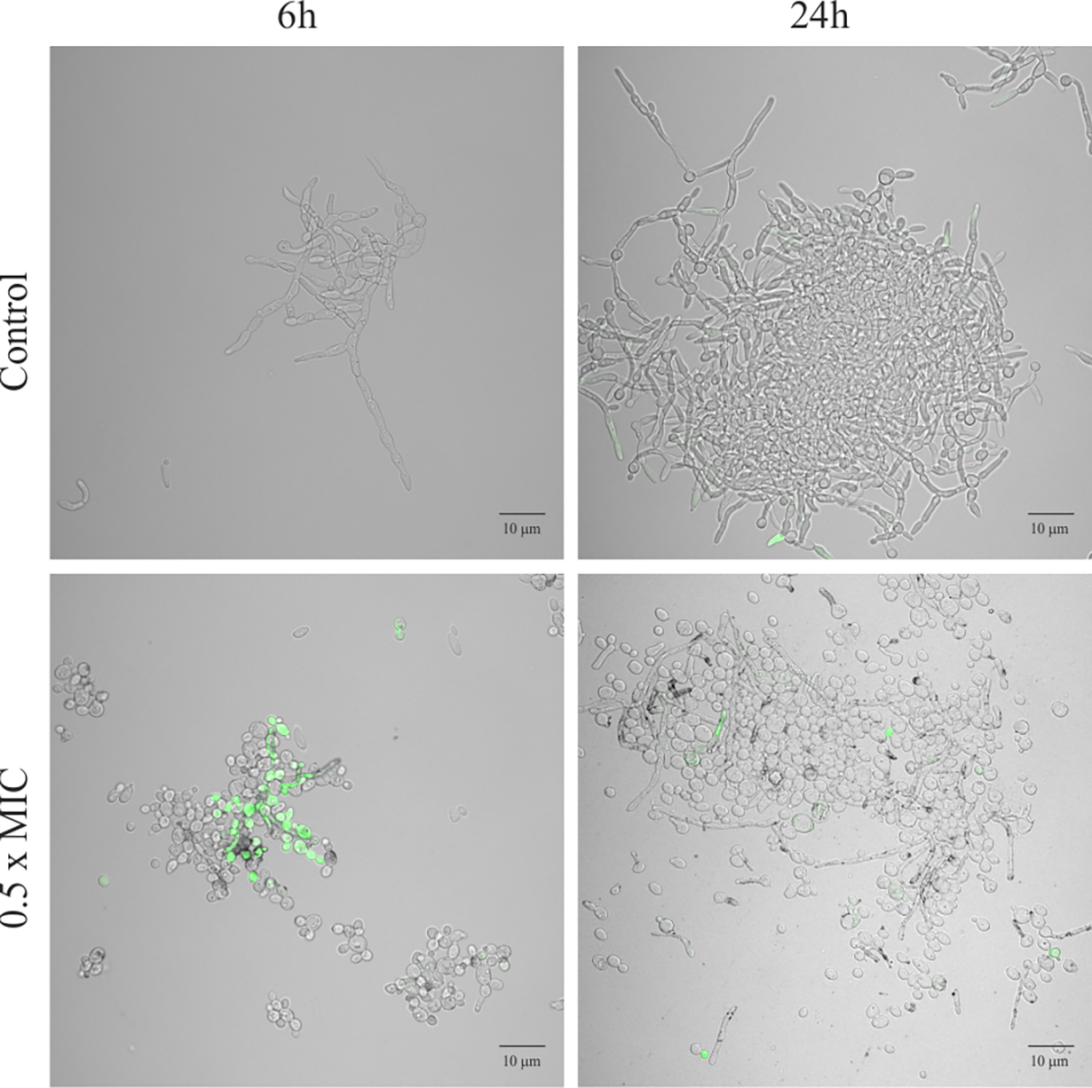
Time-dependent increase of intracellular ROS level in *C*. *albicans* ATCC 90028 treated with AgNPs at 0.5× MIC, assessed microscopically. Representative images of fields in control and tested samples. Green colored cells mean that in cells accumulating ROS, non-fluorescent H_2_DCFDA is converted to highly fluorescent 2',7'-dichlorofluorescein. Black dots show AgNPs attached to the *C*. *albicans* cells.

It has been established that intracellular release of silver ions from the nanoparticles occurs, which then interact with the thiol residues of enzymes, thereby reducing their activity [35, 36]. This led us to measure metabolic activity using FDA substrate, in parallel with the kinetics of growth (CFU). FDA, a colorless compound, is hydrolyzed by both exo- and/or membrane bound enzymes, releasing a fluorescent product, fluorescein [21]. The esterase activity up to 2 h incubation of the yeast with AgNPs at MIC or 0.5× MIC was unchanged at 92.6 and 100% of the control, respectively, whereas after 6 h it was only 5.45 and 45%, respectively. CLSM analysis of oxidative stress in *C*. *albicans* exposed to nanosilver at 0.5× MIC demonstrated the intracellular accumulation of ROS, but the effect was transient and returned in 24 h to the control level (Fig 5). In the MIC-treated cells, membrane permeability rapidly increased, and the non-compensated oxidation reaction resulted in the generation of intracellular ROS. Observations from the first 2 h of yeast exposure to AgNPs indicate that these effects escalated and were irreversible. This did not allow for a reliable microscopic imaging due to the drastic decrease in cell number.

Thus AgNPs toxicity was largely caused by the generation of ROS in target cells. Some authors [34–36] have proposed that this may partly follow a "Trojan-horse" type of mechanism when AgNPs are taken up by the cell and dissolution occurs inside the cytoplasm, causing a high local concentration of Ag^+^. There may also be external dissolution of AgNPs [36]. However, a well-characterized response of eukaryotic microbes, such as *Candida* spp., to ROS is the rapid induction of mRNA encoding oxidative stress detoxification and repair proteins. These fungi appear to induce the genes encoding antioxidants, such as catalase, glutathione peroxidase, superoxide dismutase and components of the glutathione/glutaredoxin and thioredoxin systems. The first one is critical in repairing oxidatively-damaged proteins. However, it is accepted that the mechanisms of toxicity of divalent metal ions in microbial cells are multifactorial and overlapping, and not all are clearly proven [35,37]. Increased intracellular ROS production, impaired function of metal-based antioxidant enzymes, weakening of membrane function as reflection of the lipid peroxidation are most often shown in many *in vitro* biological systems on prokaryotic and eukaryotic cells [37, 38]. The results of our research point to the importance of several of them.

**Fig 6:**
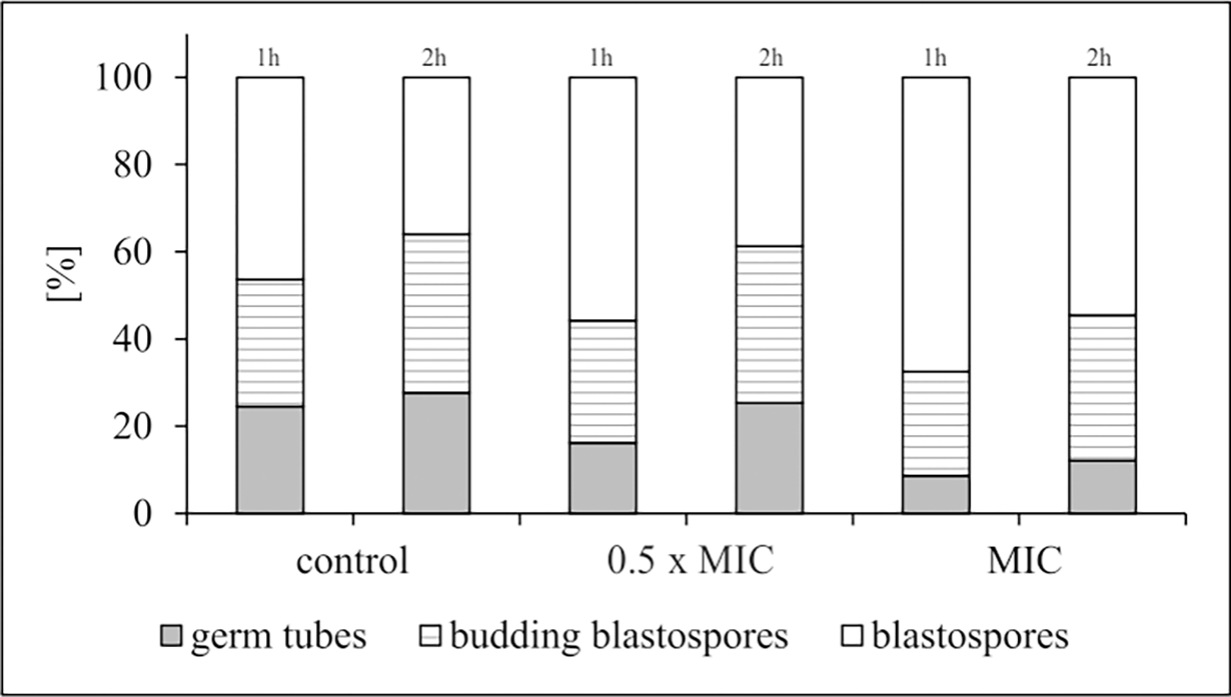
Percentage of *C*. *albicans* ATCC 90028 morphological forms after 1 and 2 h exposure to AgNPs at MIC or 0.5× MIC. *C*. *albicans* cell morphology was examined by light microscopy (400× magnification) at each time-point. The results were expressed as the proportion (± S.D.) of each morphotype after AgNPs treatment compared to control *C*. *albicans*, assessing 500 cells.

**Fig 7:**
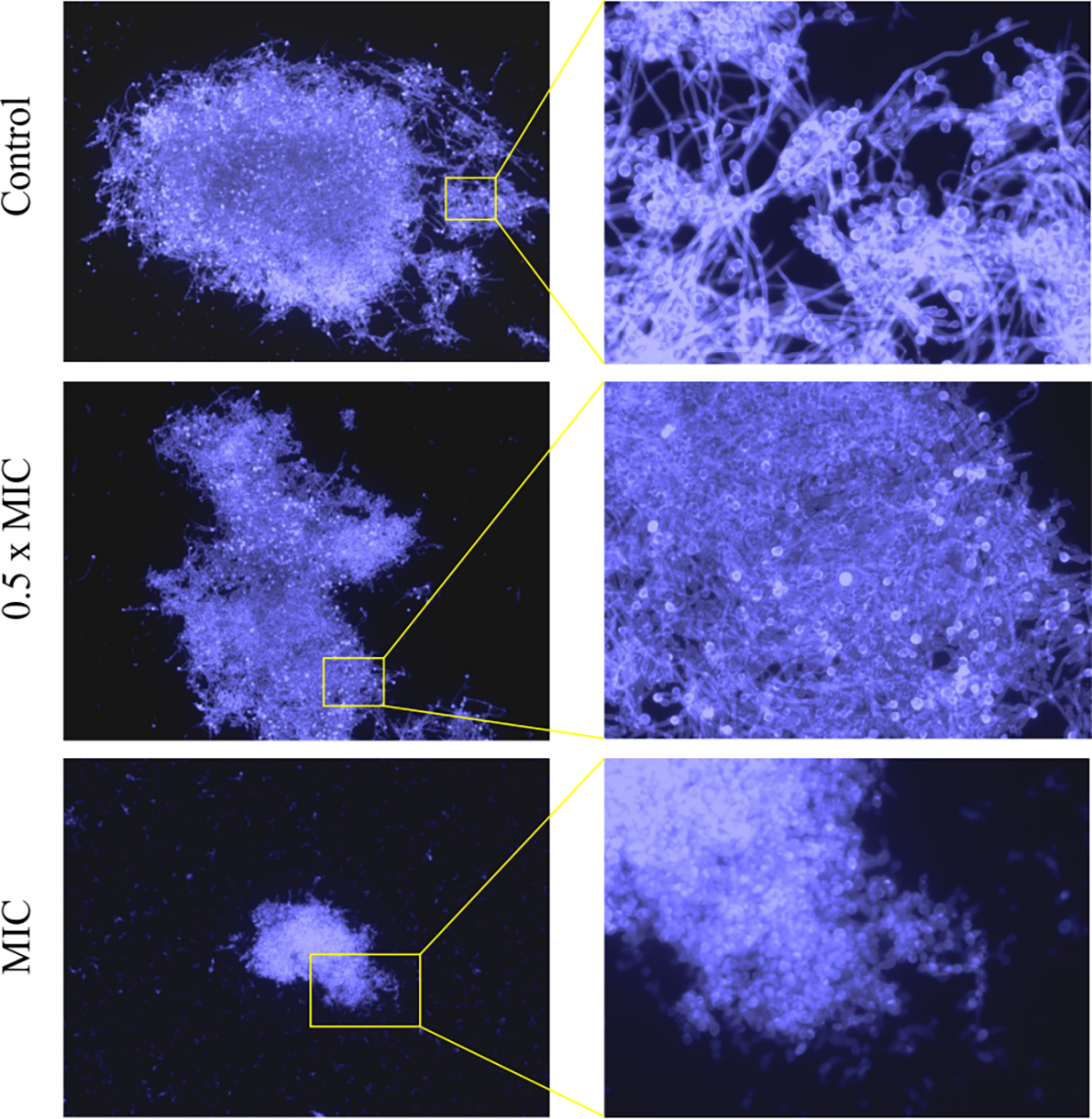
Mycelium of *C*. *albicans* ATCC 90028 formation after 24 h exposure to AgNPs at MIC or 0.5× MIC. *C*. *albicans* mycelium stained with Calcofluor White was checked microscopically (fluorescence microscope, Zeiss, AXIO Scope A1, magnification 400×); representative images are shown.

Disrupting the physiological structure and phospholipid content/proportion of *C*. *albicans* cell membrane probably disturb yeast morphogenesis. Increasing evidence over the last few decades has given important insight into filamentation as a main virulence factor contributing to candidiasis pathogenesis. Therefore, this is one of *C*. *albicans* feature to receive the most attention, and perhaps shows most promise as a target for new anti-virulence strategies [3,6,17,38], and led us to undertake experiments to specifically check this possibility. Decreased potential of filamentation and the budding process of blastospores were seen in the experiments (Figs 6 and 7). The ability of *C*. *albicans* to transform morphologically could be inhibited not only by lethal, but by sub-lethal, concentrations of AgNPs. The inhibitory effect of nanosilver was certainly achieved under optimal conditions for yeast, such as the presence of 10% serum in the culture medium, which did not weaken the anti-fungal activity of AgNPs. This is an important positive observation given that, in a protein rich environment, biomolecules form a corona around nanoparticles, which can alter their biological activity and possibly their antimicrobial or cytotoxic potential [39]. In the system studied by us, such dependence did not occur.

The nanoscale silver we tested possessed not only strong antifungal activity against free-floating cells, but had anti-adhesive and anti-biofilm potential. With the increasing threat of multidrug resistance and the shortage of new chemotherapeutic agents, there is more interest in using metals instead of, or in addition to, classical drug as a new trend in an anti-biofilm strategy [3,7,16,18]. For example fluconazole, which is widely used in the treatment of superficial fungal infections, elicited only a minute reduction in fungal adhesion and biofim formation *in vitro* even far above its MIC. By contrast, a sub-inhibitory concentration of our AgNPs (0.5× MIC = 3.125 μg/mL) reduced *C*. *albicans* adhesion to almost 38% during short-term action (2 h), as well as inhibited biofilm formation by at least 40% (during longer-lasting action, 24 h). The strong repressive effect of nanosilver on a biofilm could be due to inhibition of yeast morphogenesis, which we also noted (Figs 6 and 7). Both morphological forms (blastospores and hyphae) have a unique role in the process of *C*. *albicans* biofilm development, maturation and stability. Therefore, prevention of blastospores adhesion and differentiation into a filamentous form by AgNPs seems to offer a good therapeutic option [3, 6,16,17,38]. For many non-antibiotic products, including metal nanoparticles, two mechanisms of anti-adhesive action can be considered. The first is based on the direct biocidal activity limiting the pool of living microbial cells. The second involves inhibition of the expression of microbial adhesins and/or interference in yeast morphogenesis. This process is mediated mainly by at least 3 types of adhesins: ALS—agglutinin-like sequence adhesin family, HWP1—hyphal wall protein-1, and EAP1 adhesin, which is expressed on both morphological forms—blastospores and hyphae [16,18,27].

The high activity of NPs against the planktonic forms of *C*. *albicans*, as well as inhibition of their adherence and biofilm formation, indicated that biogenic nanosilver in our test would also be active in the eradication of a pre-formed biofilm. Currently, the most exciting observations concern the successful use of AgNPs in combating microbial biofilms on the surfaces of medical utility [40]. Indeed, some metallic nanoparticles can be active against bacterial biofilms developed on different surfaces; however, very few reports concern the control of pathogenic fungal biofilms and their co-action with anti-mycotics [41–43]. Therefore, the effect of NPs and classic drug, alone or in combination, on yeast pre-formed biofilm was assessed in further research. In this case, AgNPs were used at concentration ranging from MIC (= 6.25 μg/mL pre-determined for planktonic population) to 16× MIC. Fluconazole at MIC (= 1.0 μg/mL pre-determined for planktonic population) up to 256× MIC was used as a reference drug. The final metabolic activity of the treated biomass was measured by the Alamar blue reduction assay [44]. AgNPs were very effective biofilm-control agents; 8× MIC reduced metabolic activity of the biomass by 46.3 ± 15.0%, whereas FLC reach the MBEC_50_ level only at 64× MIC.

**Fig 8:**
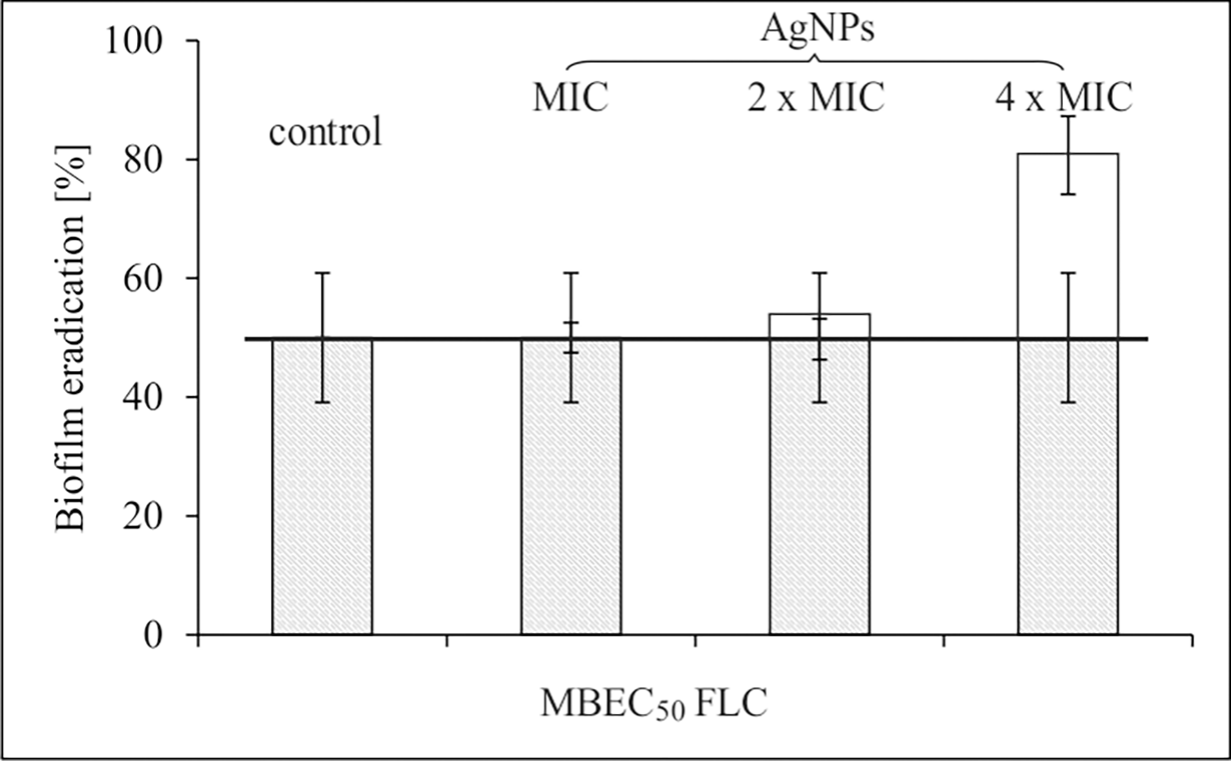
*C*. *albicans* ATCC 90028 pre-formed biofilm eradication by fluconazole at MBEC_50_ (alone, grey bars), and MBEC_50 FLC_ in combination + MIC_AgNPs_; + 2× MIC_AgNPs_; + 4× MIC_AgNPs_ (+ upper white bars). Biomass total metabolic activity was measured by the Alamar Blue-reduction assay.

The data from experiments where FLC and AgNPs were in combinations as follow: A) MIC_FLC_ + MIC_AgNPs_; + 2× MIC_AgNPs_; + 4× MIC_AgNPs_ or B) MBEC_50 FLC_ + MIC_AgNPs_; + 2× MIC_AgNPs_; + 4× MIC_AgNPs_ proved to be interesting. Version A) showed no statistically significant increase in anti-biofilm efficacy, the MBEC value of complex preparations being close to silver alone. By contrast, in version B), combination of FLC (MBEC_50_) with AgNPs significantly reduced biofilm metabolic activity. Using, as addition, 4× MIC of AgNPs eradication by exceeded the MBEC_80_ level was achieved (Fig 8).

Based on the results, it proved difficult to identify the exact mechanism responsible for any anti-biofilm synergism of fluconazole with nanosilver. However, we suggested that it may be due to the enhanced penetration of the two agents into the biofilm structure, which is sufficient to disturb the lipidome of cell membranes, and to reduce the efficacy of efflux transporters of the drug as well as to influence other anti-FLC biofilm-associated resistance mechanisms.

Nanoparticles based on silver, copper ions, and titanium, magnesium, aluminium, zinc oxides are indeed in focus as potential anti-biofilm agents. These are called metal nanobullets that have the ability to enhance antibiotic activity against a sessile population, but the question is why. It is proposed that metallic NPs easily penetrate polymeric matrix of biofilm structure, then after attaching to the surface of a single microbial cell they disrupt membranes or alter its permeability. This allows NPs to penetrate the cell and generate ROS or the released metal ion can damage macromolecules—enzymes, ribosomes, DNA and/or RNA—usually being lethal. Moreover, efflux pumps usually over-expressed in biofilms can be inhibited by different metallic NPs. The anti-biofilm activity of AgNPs could be also attributed to the extremely easy binding and penetration of these small sized, round particles to the target cell [6,7,35,36]. Depending on the metal chemistry, however, the mechanisms of activity and toxicity might differ, since they are often multifactorial and overlapping, and not all of them have so far been clearly proven. Overall, the enhanced efficacy of conventional drugs by nanoscale silver used at relatively low concentrations may be attributed to so-called "chemosensitization" effect of the target cells [1]. The main mechanisms are based on destabilization of the structural integrity of the cell membrane, disturbance in morphogenesis and disruption of the fungal stress response. These should result in an additive or synergistic therapeutic effect with classical drugs [4,5,16,20,28,42,43]. Indeed, we found that selected FLC and AgNPs combinations, measured by the checkerboard dilution method against planktonic culture, showed synergism (according to [20] FICI values from 0.3 to 0.7). Such an effect was noted for: 0.5× MIC_FLC_ + 0.06× MIC_AgNPs_ (FICI = 0.56); 0.25× MIC_FLC_ + 0.25× MIC_AgNPs_ (FICI = 0.5); 0.125× MIC_FLC_ + 0.5× MIC_AgNPs_ (FICI = 0.63). This means that there is potentially an effective therapeutic strengthening of the "old" anti-mycotic agent by the addition of relatively low concentrations of silver nanoparticles.

**Fig 9:**
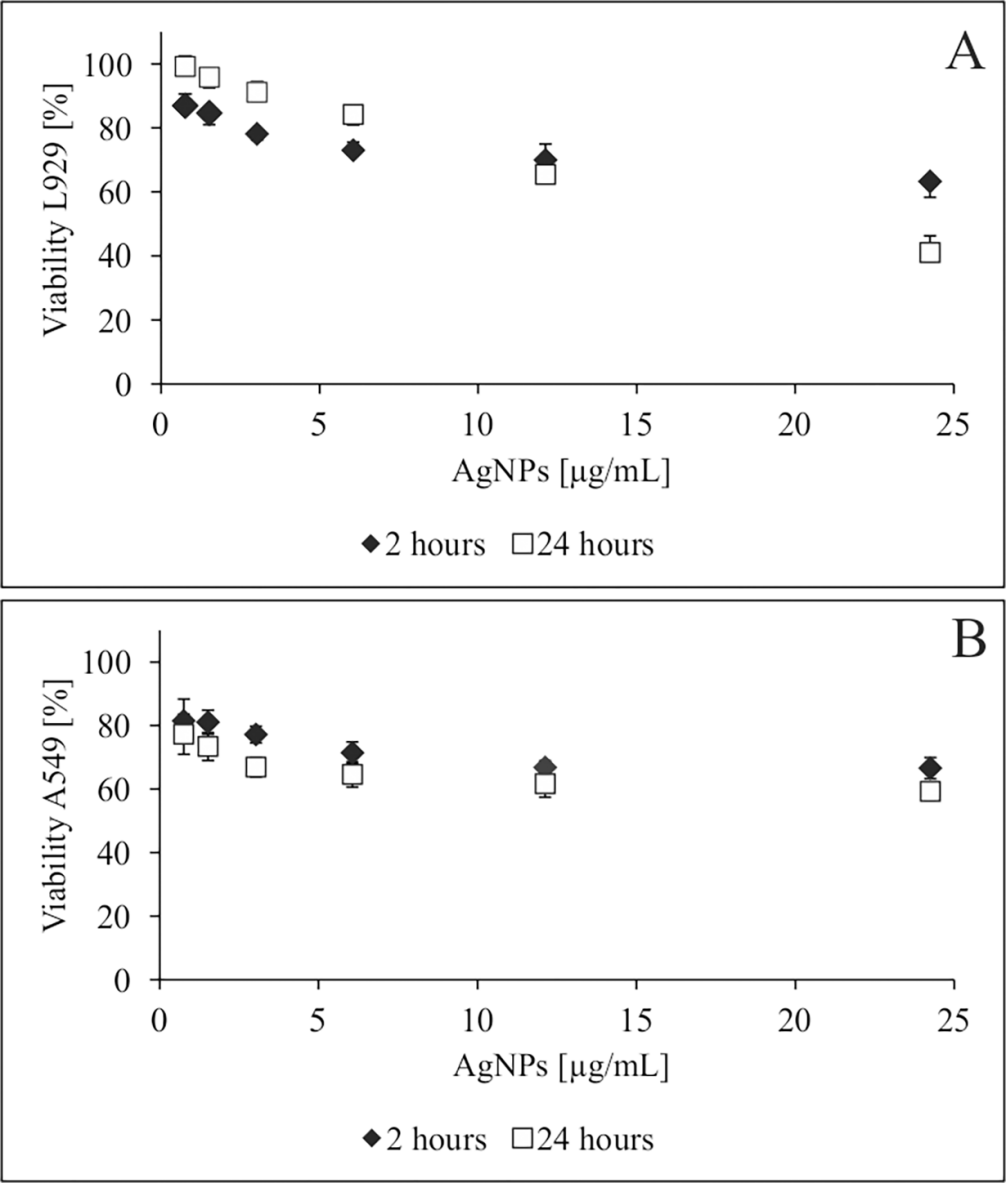
Effect of AgNPs on (A) L929 fibroblasts and (B) A549 lung epithelial cell viability using the MTT reduction assay. The percentage ± S.D. of viable cells after 2 and 24 h co-incubation compared with the viability of the control (untreated) cells taken as 100%.

However, this is not to suggest that such a combination can be useful in many different clinical situations, in which you have to be careful with possible known toxic effects of AgNPs on mammalian cells [40,43]. This is why we asked the question, is this strategy biologically safe? All materials/therapeutic products that enter the blood or other body fluids/tissues come in contact with host epithelial/endothelial cells and red blood cells in the circulation. Thus our *in vitro* studies tried to explain this issue by testing AgNPs at 0.19–25.0 μg/mL, which corresponds to 0.3× MIC to 4× MIC (for yeast). It was shown that AgNPs were non-cytotoxic (IC_50_ of 47.2 μg/mL after 2 h incubation with the L929 cell line, and 68.8 μg/mL against A549 cell line). Values of IC_50_ after 24 h of incubation were as follows: 25.5 and 22.6 μg/mL against L929 and A549 cell lines, respectively (Fig 9). To assess the effect of AgNPs on erythrocytes, hemolysis was measured as the release of hemoglobin after exposure to a range of concentrations of nanosilver, PBS and Triton-X-100 being used as negative and positive controls. Dose-dependent and time-dependent data showed no significant hemolysis. AgNPs over the range of concentrations used (including MIC, 2× MIC and 4× MIC for fungi) caused no hemolysis during 2 h of exposure. By contrast, hemolysis induced by AgNPs occurred only after 24 h incubation: 1.5% at 0.5× MIC, 2% at MIC, 5% at 2× MIC, 12% at 4× MIC. This low cytotoxicity/hemolysis of AgNPs is encouraging for the future direct application of this kind of preparation to eukaryotic tissues (e.g. in the form of surface-active ointments, lotions, dressings, or as an ingredient of antimicrobial disinfectants), since their effective anti-fungal level is lower.

In summary, research into eco-friendly tools for metal NPs synthesis and the effectiveness of their combination with antibiotics or other biocides, is widely conducted. They aim to develop new preparations for use not only in medicine but also in various biological and industry sectors, including the food industry, environment protection and agriculture [6, 9, 35, 40, 42, 43, 45]. Recently, plants and microorganisms have been shown to be good bio-factories in which NPs can be produced. Phytonanotechnology enables simple, fast and efficient method of metal NPs synthesis using aqueous extracts from various parts of plants. Such extracts contain enough proteins, amino acids, vitamins, enzymes and diverse secondary metabolites that act as reducing and capping agents, as well as stabilizing formed NPs. The mechanisms of NPs synthesis by plant species are different and not fully determined [10, 12, 45]. While, extracellular synthesis of NPs by the microorganisms (bacteria, actinomycetes, yeast, filamentous fungi) has been much better explored and shown to be more useful due to elimination of subsequent NPs purification stages. Mycosynthesis of NPs used in our study is considered better approach, because fungi compared to bacteria have higher tolerance on metals and the ability to their accumulation thus more effective NPs synthesis, utilizing mainly reductases [11, 28, 45, 46].

However, despite well known numerous advantages of nanoparticles biosynthesis, a critical look at the possible disadvantages seems to be also necessary. Many questions were asked regarding the optimization of synthesis conditions that ensure high efficiency of bionanoparticles as well as the possibilities and safety of their real application. The biggest challenge, with respect to the most commonly tested and used nanosilver, is to minimize AgNPs tendency to aggregate which can lower their biological, including microbicidal activity. It is suggested that the use of other biomolecules for the synthesis of AgNPs and others metal NPs may overcome some of these disadvantages. As reported by many authors, one of such biomolecule that can be used for the targeted synthesis of nanoparticles, is double-stranded DNA [47–51]. Based on unique dsDNA properties more complex structures (heteronanocomposites) are possible to obtain, such as DNA-templated different types of metal NPs growing on graphene oxide (GO) (Ag–GO, Au–GO, Cu–GO, Pt–GO, and Au/Cu/Pt–GO) [52]. According to the authors this future strategy allows for synthesis of NPs with good monodispersity, high stability and significant antimicrobial efficacy at relatively low concentrations It is suggested that the latter property results, among others, from the sum of the activity of both nanocomposite components—metal nanoparticles and graphene oxide [52]. Undoubtedly, continuing research in this promising direction is advisable. However, it does not lower the observations regarding the antimicrobial activity of the metal nanoparticles obtained by another synthesis route [8, 9, 35, 40, 43, 45, 53–55].

## Conclussions

Our thorough study shows that biogenic nanoscale silver altered the phenotypic behavior of *C*. *albicans*, seen as weaknesses of virulence factors essential for the development of infection. The new approach is in using AgNPs obtained by a cost-effective and environmentally friendly method. They were synthesized using an extract from the waste of filamentous fungal (*M*. *robertsii*) biomass, recovered after degradation of nonylphenol. This shows that we can achieve simultaneously two important target goals regarding their environmental and biomedical value. First, because this fungal culture can degrade nonylphenol, xenobiotic which can accumulate and persist in the environment, posing potential hazardous role as an endocrine disruptor and xeno-estrogen [15]. Second, because the biological waste (mycelium) can be re-used to obtain potentially medically useful products. The current investigations are complex in nature and include multifaceted approach to assessing antifungal activity of nanoparticles derived from "green" synthesis. These biogenic small, spherical in shape AgNPs remain stable *in vitro* for a long time and were not cytotoxic at concentrations that had a potent inhibitory effect on *C*. *albicans* multiplication, germ tube formation, hyphal growth and biofilm formation. They can also eradicate fungi within biofilms at higher efficiency than other important medications, and show synergism with fluconazole. These enables the possibility of finding future uses for the product as novel therapeutic supporting classic drugs in the course of fungal infections, at least in terms of topical therapy.
